# The effects of laser assisted hatching on pregnancy rates 

**Published:** 2011

**Authors:** Alireza Ghannadi, Marjaneh Kazerooni, Fatemeh Jamalzadeh, Sahar Amiri, Parifar Rostami, Forouzan Absalan

**Affiliations:** 1 Faculty of Nursing and Midwifery, Islamic Azad University, Larestan Branch, Larestan, Iran.; 2 Shiraz Infertility and Limited Surgical Center, Shiraz, Iran.; 3 Department of Anatomy, Medical Faculty, Ahvaz University of Medical Sciences, Ahvaz, Iran.

**Keywords:** *Laser- assisted hatching*, *Zonapellucida*, *Assistedreproductivetechnologies*

## Abstract

**Background:** For infertile women aged over 35 years, failure of the ZP (zona pellucida) to rupture is believed to be associated with a decreased implantation rate in in vitro fertilization (IVF) or intra cytoplasmic sperm injection (ICSI).

**Objective: **In this research, laser assisted hatching (LAH) was offered to patients with advanced maternal age to evaluate a possible benefit.

**Materials and Methods:** Nine hundred thirty two cycles of IVF/ICSI in females were analyzed. Women included in this study were allocated in 4 groups. In group I and II, embryos were cultured and transferred with and without LAH in women aged ≤35, whereas embryos of group III and IV were examined with and without LAH in women aged ≥ 35. Laser manipulations were performed using a suturn-Tm3 system using 2-3 pulses of 0.8 millisecond with 400 voltage duration. The size of the hole made in the zona was measured to be 5-10 µm, depending on the zona thickness of each individual embryo.

**Results:** The performance of LAH significantly increased clinical pregnancy rates in all patients. In group I and II, the chemical (50.99% and 31.61% respectively), clinical (50% and 30.69% respectively) and multiple pregnancies (22.27% and 5.94% respectively) significantly differ between these groups. In the patients with advanced female age ≥35 the performance of LAH significantly increased chemical (30.12%) and clinical pregnancy (27.71%) rates compared to whom without LAH (18.96% and 16.37% respectively).

**Conclusion:** Our data demonstrate in the patients who were less than 35 years old, multiple pregnancy rates were significantly increased compared to other groups who aged over 35 years old. In addition benefit of LAH in improving pregnancy rates after IVF or ICSI in women of advanced age (≥35) was shown.

## Introduction

Up to the blastocyst stage, the mammalian embryo is surrounded by the Zona pellucida. Prior to the implantation, the embryo has to escape from the ZP, a process known as hatching (1). Normal embryo hatching is accomplished predominantly by zona lysis and not by pressure exerted by the expanding blastocyst (2). The failure of embryo hatching may be one of the various factors that limited human reproductive efficiency, specifically in assisted reproductive technologies (ART) fields (3). Assisted hatching of human embryos before their transfer in ART program has been proposed to be beneficial, especially in cases of cultured embryos with a thick and/or dense ZP. A thick ZP may be associated with advanced woman's age (4) or poor embryo quality (5). Elasticity and thinning of the ZP are essential for the hatching process, both of which can be adversely influenced by advancing maternal age and in vitro culture conditions (6, 8). ZP is dissolved in lysine, therefore quantitative or qualities deficiencies in its secretion could result in hatching impairment (6). Suboptimal culture conditions may cause such deficiencies. The trophoectoderm of some embryos may not be able to secret the hatching factor, and lysine production could be influenced by patient's age (6, 9). It has been proposed that the artificial creation of an opening in the embryo’s zona would be a potentially useful method, which might augment the implantation rates by facilitating the hatching process, and permitted an earlier contact between the embryo and the receptive endometrium (3). The artificial gap produced by hatching may also serve as a channel for the exchange of metabolites and growth factors to and from the endometrium (6). Several methods have been employed for assisted hatching. Partial zona dissection (PZD) (9) was widely used in the beginning. Later, chemical opening by means of acidic Tyrode’s solution was performed, also known as zona drilling (10). The recent development laser system offers an excellent method for zona manipulation (11). In this system laser beams is delivered through the objective and thus can be opened zona instantaneously with a single laser plus in a few milliseconds duration.

In recent study, we investigated the benefit of laser assisted hatching in patient group at our clinic, especially couples with advanced maternal age (over 35 years of age).

## Materials and methods

In this experimental study, from September 1, 2005 to April 30, 2007 women who presented at, or were referred to, Shiraz infertility center undergoing IVF or Intra cytoplasmic sperm injection (ICSI) were included in this study. All subjects had underwent controlled ovarian hyper stimulation (with a long protocol regimen in all cases, including down-regulation with GnRH analogue and ovarian stimulation with recombinant FSH and HMG) and transvaginal oocyte retrieval after injection of 10.000 IU of HCG. In vitro fertilization or ICSI was performed with the husband’s spermatozoa according to the routine protocols at our center, and sequential culture media from vitrolife (G-IVF, G1, and G2 and Embryo glu, Sweden) were used in all cases. Women who fit the criteria were allocated into 4 groups. In group I and II (age ≤35) embryos were cultured and transferred with and without LAH, Whereas embryos of group III and IV were examined and treated with and without LAH in women with advance age (over 35). Embryos transfer was performed 2 days after retrieval. In each cycle, 2-3 embryos were transferred. Embryos with ≥50% fragmentation were not transferred. Embryos were classified according to morphological criteria as follows: grade 1, embryos had even regular, spherical blastomeres with moderate refractivity, intact zona, and no or<10% fragmentation; grade 2, embryos had uneven or irregularly shaped blastomeres, mild variation in refractivity, and<10% fragmentation; grade 3, embryos had showed<50% fragmentation and the remaining blastomeres are in a reasonable condition, with refractivity associated with cell viability, and the ZP is intact; and grade 4, when >50% of blastomeres are fragmented (12), with gross variation in refractivity and the remaining blastomeres appear viable. Laser AH was performed using the suturn-Tm3 system (Research Instrument. UK). This laser system was adapted to an inverted microscope (DMIRB, Leica, Bensheim, Germany) equipped with Hofman Modulation Contrast optics (Lieca). The diode laser beam is guided through the optical system of the microscope and can be easily focused on the target. This wavelength does not require the use of special optics or culture dishes and therefore standard culture dishes can be employed. This characteristic allow for a touch-free delivery of laser beam. For laser-drilling, culture dishes with the embryos were placed on the microscope stage. A tangential position of the zona pellucid was focused and the laser treatment was applied by using 2-3 pulses (depending on thickness of the ZP at time of ablation) of 0.8 millisecond with 400 voltage duration. The size of the hole made in the ZP was measured to be 5-10 micro meter, depending on the zona thickness of each individual embryo.

All ETs were performed after 2 days with the use of a Labotek catheter. Chemical pregnancy was defined by analyzing of β hCG hormone and clinical pregnancy as a distinct intrauterine gestational sac seen on transvaginal ultrasound.

**Statistical analysis**

Chemical and clinical pregnancy rates were analyzed on the basis of transfer cycles. Statistical analysis was performed with student’s t-test or chi- square test. In all cases, p<0.05 was considered statistically significant. 

## Results

Of the 199 patients who had over 35 years old, the chemical and clinical pregnancy rates were significantly increased in the LAH group (30.12%, 27.71%) compared to the control group (18.96%, 16.37%), (Table I). Interestingly, the chemical and clinical rates of 733 patients who had less than 35 years old were more significantly increased in the LAH group (50.99%, 50%) when compared to the control group (31.61%, 30.69%) (Table I). No significant differences were observed between multiple pregnancies rates of the two groups (13.04% in the laser group compared to 5.26% in control) in all over 35 years’ old women that were pregnant. Comparison the results in all patients under 35 years old that were pregnant, showed increase in multiple pregnancies rates in the laser group (22.27%) compared to 5.94% no LAH group (Table I). In all 932 patients of this study (under and over 35 years old) the clinical pregnancy rates showed significant difference between LAH group (46.20%) compared to no LAH group (26.96%) (Table I).

**Table I:** Subjects' characteristics and outcomes

**Characteristics**	**No. of study subjects**	**Chemical pregnancy rates (%)**	**Clinical pregnancy rates (%)**	**Multiple pregnancy rates (%)**
LAH group (>35y)	83	25 (30.12%)*	23 (27.71%)*	3 (13.04%)
No LAH group (>35y)	116	22 (18.96%)	19 (16.37%)	1 (5.26%)
LAH group (≤35y)	404	206 (50.99%)*	202 (50%)*	45 (22.27%)*
No LAH group (≤35y)	329	104 (31.61%)	101 (30.69%)	6 (5.94%)
LAH group (total )	487	-	225 (46.20%)*	-
No LAH group (total)	445	-	120 (26.96%)	-

Summarizes subjects ' characteristics and outcomes.

(*) Significant differences with no LAH group in the same age. (p < 0.05).

**Figure 1:**
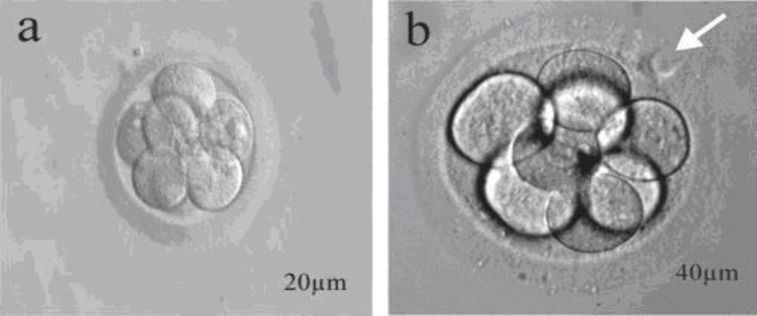
**a: **Intact 8 cells embryo, **b:** 8 cells embryo that received laser beam

## Discussion

According to previous studies advanced maternal age is one of the options that influenced the chance for embryo hatching due to the hardening of ZP (13) and that assisted hatching (AH) of the ZP in ART treatments may improve the implantation and pregnancy rates (14, 15). Assisted hatching still is a controversial issue in assisted reproduction according to patients population, operator experience, study design and the AH methods used (14). Therefore, it is difficult to compare the results described for assisted hatching from different publications. There are many investigations that shown the beneficial effects of AH on pregnancy and implantation rates in cases with advanced maternal age and previous implantation failures (16). Besides very recent studies reported that the AH does not improve implantation and clinical pregnancy in patients less than 38 years of age (17-19). According to our study, laser assisted zona thinning of human embryo before transfer has beneficial effects on clinical outcome of the two different maternal age patient population; younger and older than 35 years. It has been suggested that, in vitro culture conditions may impair the mechanism of blastocyst hatching (20, 21) due to the absence of lysine and other molecules secreted in vivo from the natural surrounding tissue, or due to the zona’s glucoprotein cross-links (4). Also, it was shown that fewer than 25% of the expanded blastocysts have been observed to hatch in vitro, presumably secondary to zona hardening (22). In contrast, embryos with a thin zona and those that have been subjected to microsurgical dissection resulting in artificial gaps in their zona were observed to implant more efficiently (23, 24). On the other hand, with LAH, there is no need to culture embryos to the blastocyst stage to increase the pregnancy rate (21). According to our study, these results showed that creating an artificial opening (slits or holes) in the ZP might improve the in vivo hatching process of embryos that are kept in culture. A similar result was obtained by other investigators that showed assisted hatching may be clinically useful in patients with a poor prognosis, including those with more than 2 failed IVF cycles and poor embryo quality and older women (≥38 years old) (25, 26). Besides, we can provide a better environment for the needs of in vitro cultured embryos, and that may be a factor eliminating the suboptimal conditions in vitro to some extent, therefore an extra zona hardening may not take place (16).

In conclusion, this research showed the benefit of LAH in improving pregnancy rates in women of advanced age (≥35) who underwent IVF or ICSI programs.

## References

[1] Drobnis EZ, Androw JB, Katz DF (1988). Biophysical properties of the ZP measured by capillary suction: is zona hardening a mechanical phenomenon?. J Exp Zool.

[2] Gordon JW, Dapunt U (1993). A new mouse model for embryos with a hatching deficiency and its use to elucidate the mechanism of blastocyst hatching. Fertil Steril.

[3] Liu HC, Cohen J, Alikani M, Noyes N, Rosenwaks Z (1993). Assisted hatching facilitates earlier implantation. Fertil Steril.

[4] Bider D, Livshits A, Yonish M, Yemini M, Mashiach S, Dor J (1997). Assisted hatching by zona drilling of human embryos in women of advanced age. Hum Reprod.

[5] Gabrielsen A, Bhantnager PR, Petersen K, Lindenberg S (2000). Influence of ZP thickness of human embryos on clinical pregnancy outcome following in vitro fertilization treatment. J Assist Reprod Genet.

[6] Cohen J, Alikani M, Trowbridge J, Rosenwaks Z (1992). Implantation enhancement by selective assisted hatching using zona drilling of human embryos with poor prognosis. Hum Reprod.

[7] Schiewe MC, Araujo E Jr, Asch RH, Balmaceda JP (1995). Enzymatic characterization of ZP hardening in human eggs and embryos. J Assist Reprod Genet.

[8] Mandelbaum J (1996). The effects of assisted hatching on the hatching process and implantation. Hum Reprod.

[9] Cohen J, Elsner C, Kort H, Malter H, Massey J, Mayer MP (1990). Impairment of the hatching process following IVF in the human and improvement of implantation by assisting hatching using micromanipulation. Hum Reprod.

[10] Gordon JW, Talansky BE (1986). Assisted fertilization by zona drilling: a mouse model for correction of oligospermia. J Exp Zool.

[11] Rink K, Delacretaz G, Salathe RP, Senn A, Nocera D, Germond M (1996). Non-contact microdrilling of mouse ZP with an objective-delivered 1.48 microns diode laser. Lasers Surg Med.

[12] Palermo GD, Neri QV, Hariparshad JJ, Dari OK, Veeck LL, Rosenwaks Z (2000). ICSI and its outcome. Semin Repord Med.

[13] Horng SG, Chang CL, Wu HM, Wang CW, Cheng CK, Huang HY (2002). Laser-assisted hatching of embryos in women of advanced age after in vitro fertilization: a preliminary report. Chang Gung Med J.

[14] Blake DA, Forsberg AS, Johansson BR, Wikland M (2001). Laser zona pellucida thinning- an alternative approach to assisted hatching. Hum Reprod.

[15] Ali J, Rahbar S, Burjaq H, Sultan AM, Al Flamerzi M, Shahata MA (2003). Routine laser assisted hatching results in significantly increased clinical pregnancies. J Assist Reprod Genet.

[16] Kutlu P, Atvar O, Vanlioglu OF (2010). Laser assisted zona thinning technique has no beneficial effect on the ART outcomes of two different maternal age groups. J Assist Reprod Genet.

[17] Hageman AR, Lawrence LT, Jungheim ES, Lanzendorf SE, Ratts VS, Odem RR (2007). Zona pellucid thickness does not predict pregnancy outcomes in patients less than 38 years of age undergoing in vitro fertilization: results from a prospective, randomized trial on assisted hatching. Fertil Steril.

[18] Hagemann AR, Lanzendorf SE, Jungheim ES, Chang AS, Ratts VS, Odem RR (2010). A prospective, randomized double-blinded study of assisted hatching in woman younger than 38 years undergoing in vitro fertilization. Fertil Steril.

[19] Valojerdi MR, Eftekhari- Yazdi P, Karimian L, Hassani F, Movaghar B (2010). Effects of laser zona thinning on vitrified-warmed embryo transfer at the cleavage stage: a prospective, randomized study. Reprod Biomed Online.

[20] Gabrielsen A, Bhantnager PR, Petersen K, Lindenberg S (2000). Influence of ZP thickness of human embryos on clinical pregnancy outcome following in vitro fertilization treatment. J Assist Reprod Genet.

[21] De Felici M, Siracusa G (1982). Spontaneous hardening of the ZP on mouse on oocyte during in vitro culture. Gamete Res.

[22] Fehilly CB, Cohen J, Simons RF, Fishel SB, Edwards RG (1985). Cryopreservation of cleaving embryos and expanded blastocysts in the human: a comparative study. Fertil Steril.

[23] Cohen J, Elsner C, Kort H, Malter H, Massey J, Mayer MP et al (1990). Impairment of the hatching process following IVF in the human and improvement of implantation by assisting hatching using micromanipulation. Hum Reprod.

[24] Cohen J (1991). Assisted hatching of human embryos. J In Vitro Fert Embryo Transf.

[25] Mansour RT, Rhodes CA, Aboulghar MA, Serour GL, Kamal A (2000). Transfer of zona-free embryos improves outcome in poor prognosis patients: A prospective randomized controlled study. Hum Reprod.

[26] Huisman GJ, Fauser BC, Eijkemans MJ, Pieters MH (2000). Implantation rates after in vitro fertilization and transfer of a maximum of two embryos that have undergone three to five days of culture. Fertil Steril.

